# A SAS Macro for Cosinor Analysis of Automated Heart Rate Data in the ICU

**DOI:** 10.1093/geroni/igaa057.679

**Published:** 2020-12-16

**Authors:** Margaret Doyle, Terrence Murphy, Melissa Knauert

**Affiliations:** Yale University School of Medicine, New Haven, Connecticut, United States

## Abstract

Cosinor analysis, developed by Franz Hallberg and colleagues in the 1960s, allows for the fitting of a cosine curve to data of known period and is frequently used in the analysis of circadian data. While software packages exist which will perform cosinor analyses, SAS has historically steered users to a reference (Ferrarra, 1993) that contains a flaw that results in miscalculation of the acrophase (peak) and nadir (minimum) values about half the time. The R package ‘cosinor’ includes the same error as the SAS reference. The macro we have written 1.) outputs a dataset with the correct values of acrophase, mesor, amplitude, nadir p-value for rhythmicity and R-squared for a curve fit to an individual’s data and 2.) plots the resulting curve and underlying data. While the macro does not allow for the simultaneous analysis of data from multiple individuals it will output the data from multiple curves so that these parameters can be used in downstream data analysis. We demonstrate the use of this macro on a dataset of automated heart rate data collected on patients in the intensive care unit. Given the growing use of automated data collection, it is vitally important that software that provides accurate statistical analysis of circadian phenomena be made widely available to clinical investigators.

